# Annually recurring erythema annulare centrifugum: a case report

**DOI:** 10.1186/s13256-015-0718-1

**Published:** 2015-10-22

**Authors:** Victor Desmond Mandel, Barbara Ferrari, Marco Manfredini, Francesca Giusti, Giovanni Pellacani

**Affiliations:** Department of Dermatology, University of Modena and Reggio Emilia, via del Pozzo 71, 41100 Modena, Italy

**Keywords:** Erythema annulare centrifugum, Type IV hypersensitivity reaction, Idiopathic disease, Seasonal recurrence, Spontaneous regression

## Abstract

**Introduction:**

Erythema annulare centrifugum is a rare cutaneous disease characterized by erythematous and violaceous annular plaques that usually involved the thighs and the legs. The eruption may be associated with an underlying disease and its accompanying characteristic symptoms. For these reasons, a full physical examination should be conducted to exclude underlying disorders. Annually recurring erythema annulare centrifugum is a rare and peculiar variant of erythema annulare centrifugum with the same clinical and histopathological characteristics. The lesions of annually recurring erythema annulare centrifugum tend to regress spontaneously after a variable period of days to months with yearly recurrence for many years.

**Case presentation:**

We present the case of a 46-year-old caucasian woman affected by annually recurring erythema annulare centrifugum, which is a peculiar form of superficial erythema annulare centrifugum. The lesions have the same clinical and histopathological characteristics of the classical superficial form of erythema annulare centrifugum and tend to regress spontaneously after a variable period of days to months. In our case, no precipitating factors were identified and no underlying diseases were found. Every year for the last 12 years the lesions started to appear in the summer months and regressed spontaneously in autumn.

**Conclusions:**

Cases of annually recurring erythema annulare centrifugum are rarely reported in the literature and generally no causative agent can be detected. The main feature of annually recurring erythema annulare centrifugum is the constant annual and seasonal recurrence of the lesions for many years.

## Introduction

Erythema annulare centrifugum (EAC) is a rare cutaneous disease characterized by an asymptomatic or pruritic eruption of variable duration that usually involves the thighs and the legs. The eruption may be associated with an underlying disease and its accompanying characteristic symptoms. For these reasons, a full physical examination should be conducted to exclude underlying disorders. Annually recurring erythema annulare centrifugum (AR EAC) is a peculiar form of superficial EAC. Christine first described this variant of EAC in 1930 but until now only few isolated reports have been published [1]. The lesions, which have the same clinical and histopathological characteristics of the classical superficial form of EAC, tend to regress spontaneously after a variable period of days to months with yearly recurrence for many years. We report the rare case of a patient affected by AR EAC.

## Case presentation

**Fig. 1:**
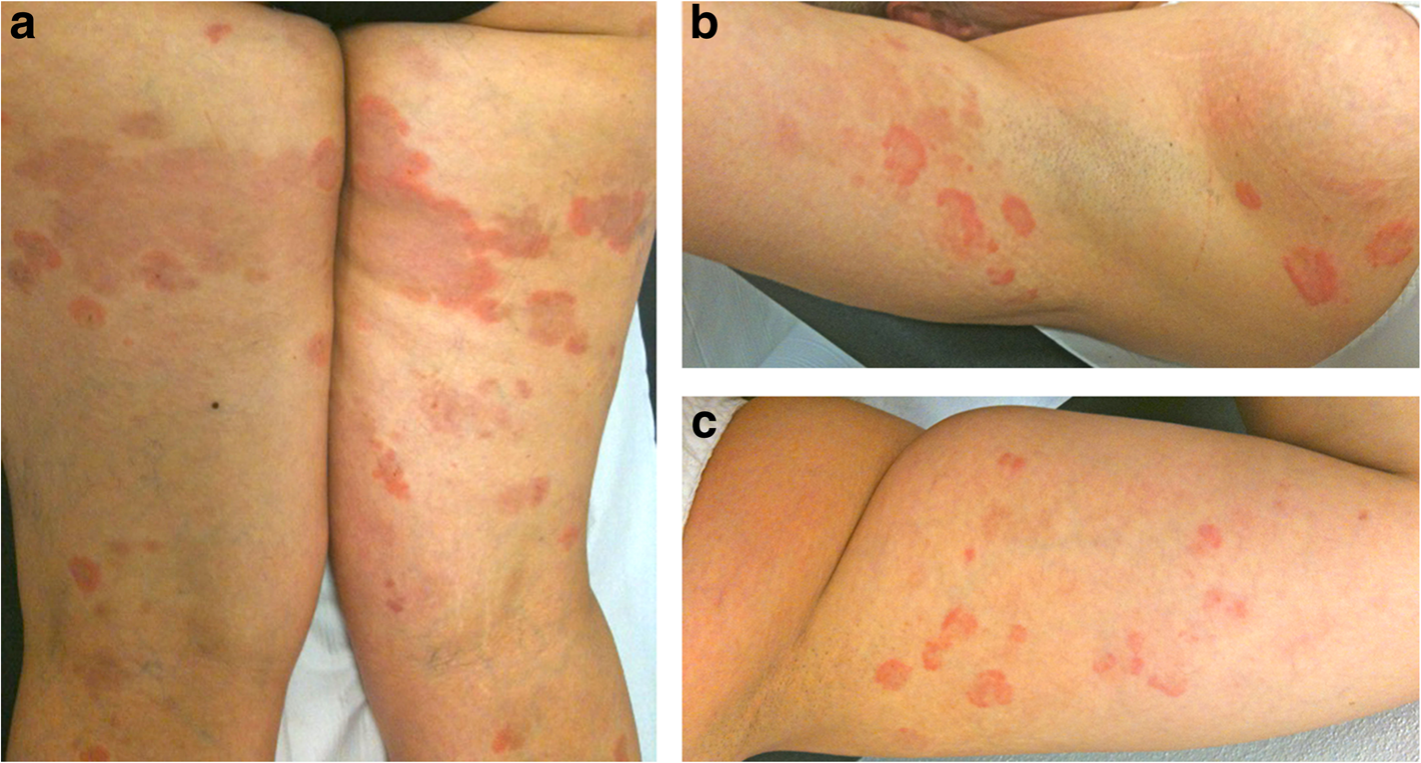
Multiple erythematous and violaceous annular plaques involving both legs (**a**) and arms (**b**, **c**). Some lesions presented a peripheral scaling border

**Fig. 2:**
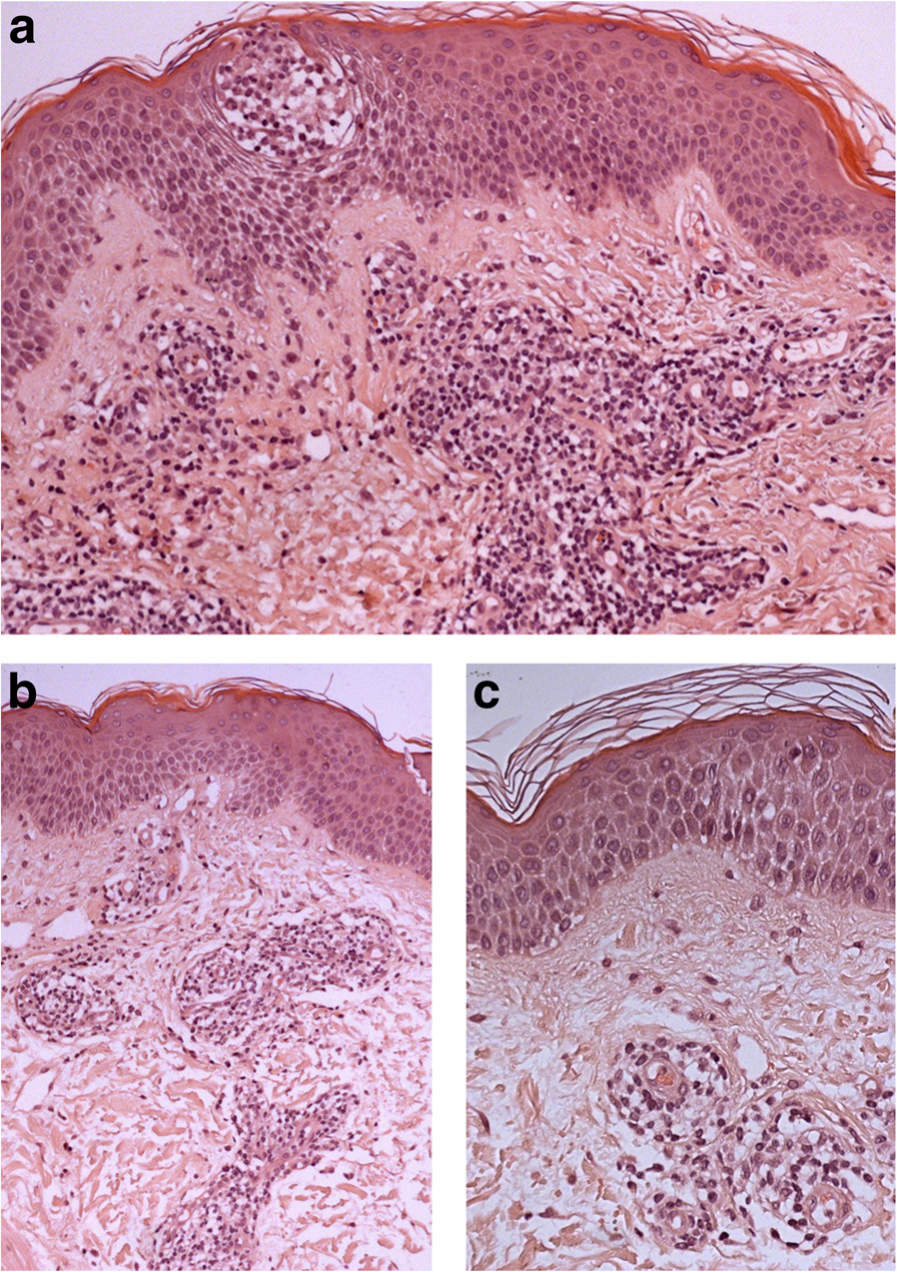
Histopathological findings of the skin biopsy showed a moderately intense superficial perivascular dermal lymphohistiocytic infiltrate with rare eosinophils, edema of papillary dermis, hyperkeratosis and focal epidermal spongiosis. Hematoxylin and eosin stain, original magnification: ×10 (**a**), ×20 (**b**), ×40 (**c**)

A 46-year-old caucasian woman was referred to our department for evaluation of a relapsing self-healing annular eruption involving her extremities that had recurred yearly for the last 12 years. A physical examination revealed multiple erythematous and violaceous annular plaques involving both legs and arms (Fig. 1). The eruption began as small erythematous papules that coalesced into annular plaques with central clearing and centrifugal spread. Our patient had reported that the lesions were intensely itchy. Some lesions presented a peripheral scaling border. Her face, hands, foot and trunk were spared. Our patient reported that the lesions began to appear every year in the summer months and regressed spontaneously in autumn. No mucosal lesions were present and the rest of the physical examination disclosed no abnormalities. Her medical history was unremarkable and our patient did not use drugs. No precipitating factors were identified. We performed a complete routine laboratory investigation including immunologic tests (immunoglobulins, rheumatoid factor, antinuclear factor and organ-specific antibodies) and all values were within the normal range. *Borrelia burgdorferi* antibodies and viral serological tests gave negative results. A potassium hydroxide microscopic slide was prepared, which yielded a negative result for a fungal infection. Her chest radiograph and mammogram were normal. Her Pap test result was negative. A skin biopsy was performed and the histologic examination revealed a moderately intense superficial perivascular dermal lymphohistiocytic infiltrate with rare eosinophils and focal epidermal spongiosis (Fig. 2). Direct skin immunofluorescence test results were negative. The clinical and histopathological features, with a supportive history of recurrent lesions, led to the diagnosis of AR EAC. The lesions regressed spontaneously 4 months after onset.

## Discussion

EAC is a rare cutaneous disease characterized by annular erythematous lesions. Generally, it can appear as a multiple polycyclic lesions simulating urticaria papules that enlarge centrifugally with central clearing. EAC was first described by Darier in 1916, and classified in 1978 by Ackerman into a superficial and a deep type [2, 3]. The superficial type is characterized by perivascular lymphohistiocytic infiltrate of variable intensity in the papillary and middermis with occasional eosinophils. Edema of the papillary dermis is usually present. The epidermal changes of parakeratosis and spongiosis may be present. Clinically, these lesions have nonindurated borders, are scaly and pruritic. Instead, the deep type is characterized by perivascular lymphohistiocytic infiltrate of variable intensity in the superficial (papillary and middermis), but in addition the inflammatory infiltrate involves the deep dermis (reticular dermis). No epidermal changes are generally observed. Clinically, these lesions have indurated borders, are nonscaly and nonpruritic. The etiology and pathogenesis are unknown [4]. It is believed that EAC represents a cutaneous manifestation of a type IV hypersensitivity reaction to different causes and underlying systemic diseases, including: food allergy, arthropod bites, drug reactions (finasteride, chloroquine, hydroxychloroquine, hydrochlorothiazide, piroxicam, etizolam, cimetidine, penicillin, salicylates, spironolactone, gold sodium thiomalate, amitriptyline, ustekinumab, rituximab), infections disease (bacterial, viral, parasitic, fungal, mycobacterial), endocrine and immunological disorders (menstrual cycle, Graves disease, Hashimoto thyroiditis, Sjögren syndrome, autoimmune progesterone dermatitis), hematological and other neoplastic disorders (Hodgkin lymphoma, non-Hodgkin lymphoma, acute leukemia, histiocytosis, multiple myeloma, nasopharyngeal carcinoma, prostatic adenocarcinoma, breast carcinoma, ovarian carcinoma) [5–10]. Treatment and eradication of the underlying disease often resolves EAC. The prognosis for EAC is excellent, except when associated with an underlying malignancy and other systemic disease. It is important to highlight that EAC can appear many years before, concomitantly or after the onset of a malignancy. For all these reasons, a diagnosis of EAC should be followed by a full physical examination and diagnostic workup in order to exclude an underlying disorder. However, sometimes no causative agent can be identified and in these cases EAC is considered idiopathic. Our case represents the peculiar variant of AR EAC, which is only exceptionally reported in the literature [1, 4, 11]. This variant has the same clinical and histopathological features of the classical superficial form of EAC and is usually observed in women (2:1). The average age of onset is 49 years; however, ages can range from 16 to 83 years [11]. The lesions tend to involve the legs and arms and sporadically the trunk, while the face, hands and foot are always spared. The lesions, which appear constantly during the spring or summer months, tend to regress spontaneously after a variable period of days to months with yearly recurrence for many years. Normally, no causative agent can be detected and for this reason AR EAC is usually considered an idiopathic disease. Moreover, associated symptoms are generally not present. Nevertheless, a full physical examination and diagnostic workup is very important in order to exclude an underlying disorder. Until now no effective treatment has been described. Photosensitivity is a tempting theory but the presence of lesions in nonexposed skin areas and the absence of facial and trunk involvement seem to rule out the role of sun exposure. It could be hypothesized that environmental factors (temperature, seasonal plants or fungus) explain the periodic course of the disease, but further studies would be necessary.

## Conclusions

AR EAC is a peculiar form of superficial EAC and until now only few isolated reports have been published. AR EAC is generally considered an idiopathic disease because no causative agent can be detected. The main feature of AR EAC is the constant annual and seasonal recurrence of the lesions for many years.

## Consent

Written informed consent was obtained from the patient for publication of this case report and accompanying images. A copy of the written consent is available for review by the Editor-in-Chief of this journal.

## Abbreviations

AR EAC: annually recurring erythema annulare centrifugum
EAC: erythema annulare centrifugum
